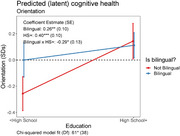# Bilingualism as a Unique Resource for Cognitive Aging

**DOI:** 10.1002/alz70860_105106

**Published:** 2025-12-23

**Authors:** Kristine J Ajrouch, Toni C Antonucci, Simon Brauer, Wassim Tarraf, Laura B. Zahodne

**Affiliations:** ^1^ University of Michigan, Ann Arbor, MI, USA; ^2^ Wayne State University, Detroit, MI, USA

## Abstract

**Background:**

While the act of immigration can involve stress for first and later generation immigrants, it might also be cognitively stimulating as it makes people experience new physical environments, new social conditions, new languages and new habits. Becoming fluent in the host country language yet retaining fluency in the language of origin may provide cognitive benefits, including buffering the negative impact of educational disadvantage. This study examines associations between education, bilingualism, and multiple domains of cognitive health among Middle Eastern and North African (MENA) older adults in the United States.

**Method:**

Data were drawn from the Detroit‐Aging and Memory Project, which included 375 MENA adults aged 65 years and above in the metro‐Detroit area who completed social, cultural and cognitive assessments. Structural Equation Modeling is used to test links between education level, bilingualism and five latent cognitive factors, adjusting for age, gender and language of interview.

**Result:**

The mean age is 73 years old. Forty‐seven percent (47%) are female and 59% have a high school education or more. More than half of the sample reported being able to speak both Arabic and English well or very well (58%) and 35% completed the assessment in English. Being bilingual was associated with better executive functioning, visuo‐spatial and orientation, but not language or episodic memory. Further, bilingualism attenuated associations between education level and cognitive health. Among those with less than a high school education, those who were bilingual exhibited higher executive function, language, visuo‐spatial and orientation scores than those with less than a high school education and not bilingual. Notably, bilingual participants with less than high school education exhibited orientation scores on par with their counterparts with high school education or more.

**Conclusion:**

Bilingualism may represent a unique resource that stimulates brain activity in lieu of lower educational experiences for minoritized ethnic and immigrant populations. Future studies should investigate whether simultaneously retaining aspects of the home culture while acquiring advantageous aspects of the host culture leads to better cognitive outcomes. Study of cognitive activities as a protective factor for cognitive health would benefit from investigations of bi‐cultural experiences.